# Differential Responses to Woodland Character and Landscape Context by Cryptic Bats in Urban Environments

**DOI:** 10.1371/journal.pone.0126850

**Published:** 2015-05-15

**Authors:** Paul R. Lintott, Nils Bunnefeld, Jeroen Minderman, Elisa Fuentes-Montemayor, Rebekah J. Mayhew, Lena Olley, Kirsty J. Park

**Affiliations:** 1 Biological and Environmental Sciences, School of Natural Sciences, University of Stirling, Stirling, Scotland; 2 Biological and Environmental Sciences, School of Natural Sciences, University of Stirling, Stirling, Scotland; 3 School of Biology, University of St Andrews, St Andrews, Scotland; 4 Biological and Environmental Sciences, School of Natural Sciences, University of Stirling, Stirling, Scotland; 5 Biological and Environmental Sciences, School of Natural Sciences, University of Stirling, Stirling, Scotland; 6 Biological and Environmental Sciences, School of Natural Sciences, University of Stirling, Stirling, Scotland; 7 Biological and Environmental Sciences, School of Natural Sciences, University of Stirling, Stirling, Scotland; Università degli Studi di Napoli Federico II, ITALY

## Abstract

Urbanisation is one of the most dramatic forms of land use change which relatively few species can adapt to. Determining how and why species respond differently to urban habitats is important in predicting future biodiversity loss as urban areas rapidly expand. Understanding how morphological or behavioural traits can influence species adaptability to the built environment may enable us to improve the effectiveness of conservation efforts. Although many bat species are able to exploit human resources, bat species richness generally declines with increasing urbanisation and there is considerable variation in the responses of different bat species to urbanisation. Here, we use acoustic recordings from two cryptic, and largely sympatric European bat species to assess differential responses in their use of fragmented urban woodland and the surrounding urban matrix. There was a high probability of *P*. *pygmaeus* activity relative to *P*. *pipistrellus* in woodlands with low clutter and understory cover which were surrounded by low levels of built environment. Additionally, the probability of recording *P*. *pygmaeus* relative to *P*. *pipistrellus* was considerably higher in urban woodland interior or edge habitat in contrast to urban grey or non-wooded green space. These results show differential habitat use occurring between two morphologically similar species; whilst the underlying mechanism for this partitioning is unknown it may be driven by competition avoidance over foraging resources. Their differing response to urbanisation indicates the difficulties involved when attempting to assess how adaptable a species is to urbanisation for conservation purposes.

## Introduction

Urbanisation is one of the most dramatic forms of land use change. By 2050 it is expected that 70% of the world’s population will live in urban areas, this expansion will require rapid urban growth which can fragment, destroy or degrade existing natural ecosystems [1]. This can lead to reductions in species richness, diversity, and changes in community composition within the urban landscape (e.g. [2], [3]). We know relatively little about the underlying mechanisms that make certain species adept at adapting to urbanisation which makes the development of management plans to conserve native biodiversity difficult to formulate [4]. Morphological or behavioural factors influence how species respond to the urban landscape, and these traits have been used to classify species as ‘urban avoiders’, ‘urban utilizers’ or ‘urban dwellers’ [5], although in reality there is likely to be a continuous spectrum of adaptability. Understanding where along this spectrum a species lies will help determine the extent of conservation action required.

The prevalence of many species within the urban environment depends on their ability to survive and adapt to heavily modified landscapes and anthropogenic disturbances. In this regard, Chiroptera are one of the few orders of animals in which many species have formed strong associations with human-modified habitats. Human habitations provide roosts, while adaptations of the environment provide food sources, such as ‘light-attracted’ bat species exploiting insect congregations that form at artificial light sources [6]. However, whilst many species have adapted to exploit the urban landscape, the general pattern is of lower bat activity and species richness with increasing levels of urbanisation (e.g. [7], [8], but see [9]). Adaptation to the built environment is highly species-specific, for example species with high mobility (e.g. those with fast, high flight) are often able to utilise habitat patches of high foraging potential in an otherwise unsuitable landscape as their movement is relatively independent from structural features. In contrast, slow flying bats may respond more strongly to small-scale features (e.g. road networks) and therefore their ranging ecology and habitat selection may be more heavily impacted [10]. For example, in the Eastern pipistrelle (*Pipistrellus subflavus)*, the location of foraging sites is influenced more strongly by the distance to hibernacula than the level of urbanisation or degree of woodland fragmentation [11].

Woodland is widely regarded as primary foraging habitat for a range of bat species [12], however urban woodland is of variable quality, subject to invasive species encroachment and often consists of small, fragmented patches [13]. Although management strategies for the conservation of urban woodland are being developed in many countries due to the benefits for human health [14] and biodiversity conservation [15] [16], their effectiveness for the latter is unknown as basic ecological data is lacking for many taxa in urban landscapes. Grouping the conservation requirements of morphologically similar species together would increase the efficiency and effectiveness of management strategies as a greater number of species would benefit from any single conservation action. However, this is problematic if morphologically similar species differ substantially and unpredictably in their response to changes or pressures associated with urbanisation.

In this paper we examine whether closely related species can respond differently to urbanisation. We use two, often sympatric, cryptic species of pipistrelle bat *P*. *pygmaeus* and *P*. *pipistrellus* which are widespread throughout Europe to investigate how habitat selection within the built environment varies between species. These two species have very similar flight morphologies [17], although they show a small but significant difference in their body size [18] and echolocation call frequencies. Little is known of the response of these cryptic species to the urban landscape although Hale et al. [19] found that *P*. *pipistrellus* activity at urban ponds peaked with moderate levels of adjacent urban grey space. Morphological traits are often linked to habitat specialisation and from this the risk of exclusion from highly modified landscapes can be inferred [20]. Consequently, the morphological similarities between *P*. *pipistrellus* and *P*. *pygmaeus* suggest that both species will respond similarly to the urban matrix. Specifically, we address the following three questions:

1. Do *P*. *pipistrellus* and *P*. *pygmaeus* respond similarly to urban woodland vegetation character (e.g. tree density) and patch configuration (woodland size and shape)?
2. Do *P*. *pipistrellus* and *P*. *pygmaeus* respond similarly to the composition, spatial configuration, and heterogeneity of the surrounding landscape and, if so, at what spatial extent?
3. Do *P*. *pipistrellus* and *P*. *pygmaeus* exhibit similar habitat selection within the urban matrix?
4. What are the conservation implications of these findings?

## Materials and Methods

### Ethics Statement

The landowners gave permission for access to all survey sites. All UK bat species are protected and licenses are required if they are to be handled or trapped, however as this study passively monitored their foraging activity there were no licensing issues. The surveying methodology was approved by the Biological and Environmental Sciences ethics committee at the University of Stirling.

### Site selection

**Fig 1:**
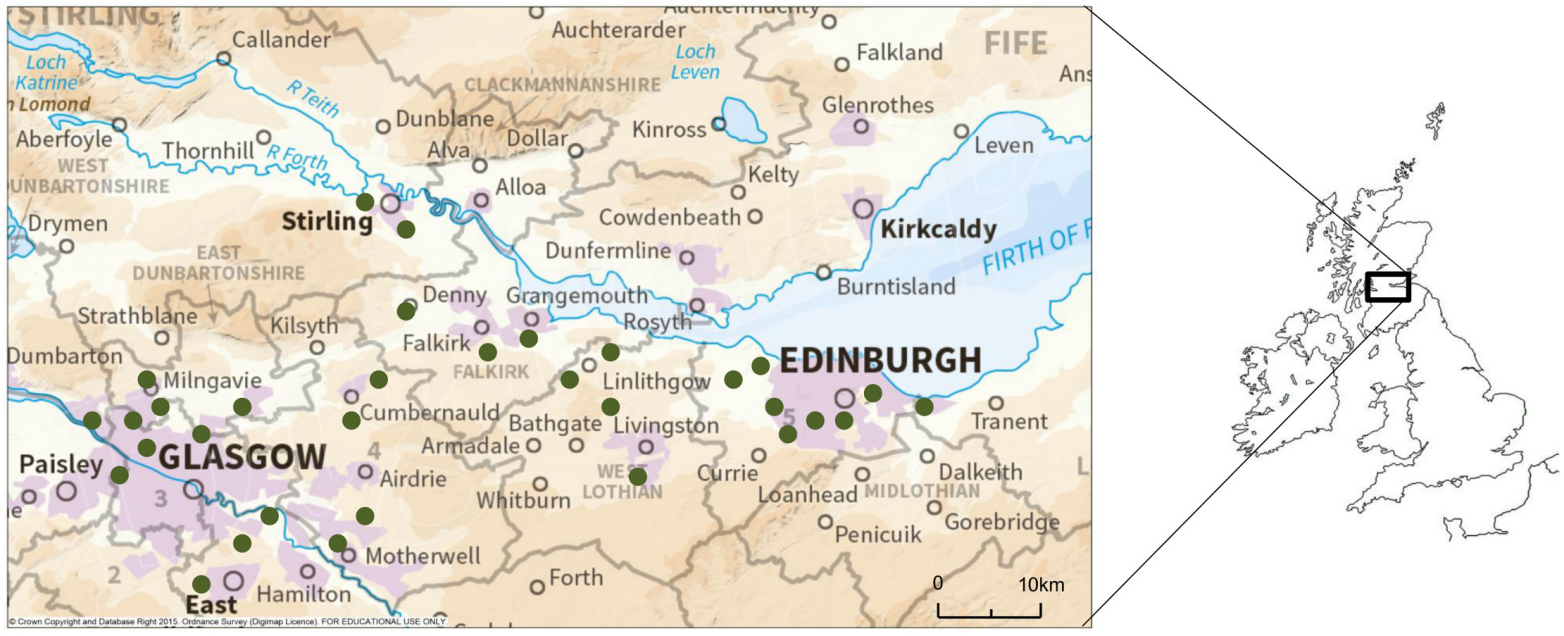
Map of central Scotland showing approximate locations of woodland sites (dark green dots) surveyed in 2011. Map produced using EDINA Digimap Ordnance Survey Service.

A total of 31 urban woodlands in central Scotland, UK (Fig 1; Table 1) were identified using Ordnance Survey digital maps [21] and surveyed between May 19th and September 1st 2011. Urban areas were designated as those where urban cover was the dominant land use within a 1 km grid square as categorised by the Centre for Ecology and Hydrology Land Cover Map 2000. Sites were selected by size, longitude, and degree of urbanisation in the surrounding 1 km using a stratified random sampling method. Selected woodlands were a minimum of 50 years old, and were either broadleaved or consisted of a mixture of conifer and broadleaved trees. Sites were surveyed in random order through the field season to avoid any spatial or temporal bias.

**Table 1:** The latitude and longitude of the 31 urban woodlands surveyed throughout central Scotland in 2011.

Survey Order	Date of Survey	Urban Area	Latitude	Longitude
1	19/05/2011	Bo'ness	56.008286	-3.593152
2	26/05/2011	Glasgow	55.945677	-4.322135
3	30/05/2011	Grangemouth	56.015572	-3.742894
4	06/06/2011	Edinburgh	55.954437	-3.254571
5	07/06/2011	Edinburgh	55.928337	-3.286886
6	27/06/2011	Coatbridge	55.854420	-3.994013
7	28/06/2011	Glasgow	55.898045	-4.315760
8	01/07/2011	Falkirk	55.990887	-3.724633
9	03/07/2011	Glasgow	55.821609	-4.061669
10	04/07/2011	Glasgow	55.837875	-4.350178
11	10/07/2011	Denny	56.012753	-3.907732
12	11/07/2011	Edinburgh	55.926800	-3.137069
13	12/07/2011	Edinburgh	55.902168	-3.24676
14	13/07/2011	Glasgow	55.915882	-4.314108
15	18/07/2011	Glasgow	55.898045	-4.315760
16	22/07/2011	Edinburgh	55.923968	-3.226897
17	24/07/2011	Glasgow	55.837170	-4.173989
18	25/07/2011	Glasgow	55.775605	-4.345211
19	26/07/2011	Livingston	55.900280	-3.524455
20	29/07/2011	Edinburgh	55.963467	-3.290362
21	30/07/2011	Glasgow	55.898685	-4.225316
22	03/08/2011	Stirling	56.098759	-3.919222
23	15/08/2011	Falkirk	55.998913	-3.743699
24	17/08/2011	Glasgow	55.885082	-4.344406
25	18/08/2011	Glasgow	55.918975	-4.204876
26	20/08/2011	Glasgow	55.927323	-4.320662
27	22/08/2011	Edinburgh	55.940029	-3.078446
28	23/08/2011	Edinburgh	55.920235	-3.195734
29	30/08/2011	Cumbernauld	55.957981	-3.977566
30	31/08/2011	Glasgow	55.817329	-4.245486
31	01/09/2011	Stirling	56.110161	-3.948866

### Vegetation surveys

Daytime vegetation surveys were conducted within a week of the bat survey to ensure that appropriate vegetative conditions were recorded. Four circular plots with radii of 20m were randomly located within each woodland patch. At each of the four plots, all trees were counted, identified to at least genus level, and tree basal area measured. Vegetation clutter was measured from 0–4 metres in height at 18 evenly spaced points within each plot to determine vertical forest structure; adopting a similar approach to Smith & Gehrt [22], a four metre pole with sixteen 0.25 subsections marked upon it was placed at each point within the plot. Any foliage, branches, or stems touching a subsection was counted and summed to provide a measure of clutter (100% clutter occurred when foliage touched all points on the pole in each of the 18 points within the plot). Within each plot canopy cover (%) was assessed at 18 points in each plot using a sighting tube with an internal crosshair; if the crosshair intersected canopy vegetation, presence of canopy was recorded. Data for the four vegetation plots were combined to provide a description of each woodland patch. Additionally, the remaining woodland was visually assessed to ensure that the vegetation surveys were representative of the entire woodland patch.

### Bat surveys

#### Woodland stand survey

Acoustic surveys were used to determine species presence and a measure of relative activity within each woodland patch. Acoustic surveys were undertaken to quantify foraging activity of bats; these are widely used in studies to determine species presence and habitat use for bats (e.g. [23, 24]), and there is evidence that pipistrelle spp. activity correlates positively with density estimates within woodland [25]. Bat activity was quantified using a frequency division bat detector (Anabat SD1, Titley Electronics) fixed on a 1 m high pole with the microphone pointing upwards. The bat detector was placed within the centre of one of the four plots (see section ‘Vegetation surveys’) and rotated between plots every 30 minutes for four hours in total (the length of the shortest night in the study area). Plot locations were ≥ 20 m from the woodland edge, and ≥ 40 m from each other and positioned to avoid paths. All bat recordings were analysed using Analook W [26]. One bat pass was defined as at least two echolocation calls within one second of each other [27, 28]. Both *P*. *pipistrellus* and *P*. *pygmaeus* can be determined by the characteristic frequency (Fc = the frequency at the right hand end of the flattest portion of a call; [26]) of their search-phase echolocation calls. Bat passes with a Fc of between 49 and 51 kHz were classed as unknown *Pipistrellus* species.

#### Urban matrix survey

Point counts (8 minute duration) were conducted at different locations within and around each woodland patch using a frequency division bat detector (Anabat SD2, Titley Electronics) to compare differences in *P*. *pipistrellus* and *P*. *pygmaeus* activity at the woodland interior, edge, surrounding green space, and grey space. Grey space was categorised as land that is sealed, impermeable ‘hard’ surfaces such as tarmac or concrete (e.g. car parks, urban housing), whilst unsealed, permeable ‘soft’ surfaces such as soil and grass were classed as non-wooded green space (e.g. parkland, amenity grassland; following [29]). Three point counts were conducted within each habitat (a total of 12 point counts per night). These were conducted simultaneously to the woodland stand survey and recordings were analysed in the same manner.

### Landscape analysis

Bat detector locations were plotted using ArcGIS 10 [30] and the centre point of the four plots within each site determined. Buffers of 250 m, 500 m, 1000 m, 1500 m, 2000 m, 2500 m and 3000m radius were created around the central point. We selected these different scales because the smallest represents site-specific characteristics, the intermediate scales have previously been found to be important predictors of pipistrelle spp. activity within human-disturbed landscapes [31], and the largest scale reflects the upper limit of home range size for *P*. *pygmaeus* and *P*. *pipistrellus* [32]. Data from the OS MasterMap Topography Layer [21] was used to reclassify the landscape within each buffer into a set of discrete biotope types. These were (i) greyspace (buildings, structures, roads, and paths); (ii) green space (gardens, parkland, managed grassland, rough grassland, and farmland); (iii) inland fresh water and (iv) woodland (coniferous, deciduous and mixed woodland). Woodland Euclidean nearest neighbour distance (ENN, the mean value of ENN distances between all woodland patches within the landscape) and the Shannon diversity index (SHDI, a measure of landscape heterogeneity) were calculated as previous studies have found these variables to influence bat foraging activity [24]. The proportion of land covered by each biotope, woodland ENN, and SHDI were calculated for each buffer scale using Fragstats v4.0 [33].

### Data analysis

Statistical analyses were undertaken using R version 2.14 [34] using the lme4 [35] and effects package [36].

### Woodland stand survey

We performed a Generalised Linear Mixed-Effects model (GLMMs; [37]) with binomial error distribution and a logit link to quantify the influence of woodland characteristics and landscape metrics on *P*. *pipistrellus* and *P*. *pygmaeus* activity. In order to assess the relative effects of these variables on *P*. *pygmaeus* in comparison to *P*. *pipistrellus*, the model was run with the proportion of *P*. *pygmaeus* to *P*. *pipistrellus* passes per plot (n = 124) as the response variable, with ‘site’ (woodland) included as a random (grouping) factor (n = 31) to account for pseudoreplication of multiple recordings per site [37, 38]. Based upon the scientific literature on the ecology of woodland bats (e.g. [24]) the following predictor variables were included in the model: (i) woodland vegetation characteristics: tree species richness, average tree basal area, woodland clutter and woodland canopy cover (covariates) and woodland type as a fixed factor; (ii) patch configuration: woodland size, woodland shape (covariates), and the interaction between size and shape. Woodland shape is the perimeter divided by the minimum perimeter possible for a maximally compact patch of the same area. This equals 1 when the patch is maximally compact and increases as shape becomes irregular [33]; (iii) landscape metrics (covariates). Temperature and date were also included in all models as covariates. Given the high collinearity found between landscape metrics (i.e. between the proportions of different biotope types or the same biotope type at a variety of spatial scales) preliminary analyses were conducted to determine which landscape metrics should be included in the model. We used GLMMs for the proportion of *P*. *pygmaeus* to *P*. *pipistrellus* passes per plot with single landscape parameters (at each spatial scale) as a preliminary assessment of which key landscape predictors should be included in the final model (i.e. highest R^2^ value). If several landscape parameters were of equal importance (i.e. <5% difference between the highest R^2^ value) they were all selected, providing they were not strongly correlated.

We present the result of the full model including standardised parameters and confidence intervals for all explanatory variables. Inferences on the effect of each parameter were made by (i) comparing its standardized estimate with other predictor variables to determine relative importance, (ii) the upper and lower 95% quantiles of each parameter distribution obtained from N = 2000 simulated draws from the estimated distribution [39], and (iii) a comparison of models excluding each parameter in turn using Likelihood Ratio Tests (LRTs) [40]. LRTs of main effect parameters also involved in interactions were performed by comparing a model excluding the main effect term to a model including all main effects (but not interactions) only. Prediction plots were constructed by undertaking simulated draws (n = 2000) from the estimated distribution of one explanatory variable whilst maintaining all other parameters in the model at their median observed values.

### Urban matrix survey

Generalised Linear Mixed-effects Models with a binomial distribution were conducted to assess differences in bat activity between habitats within the urban matrix (n = 93 per habitat); woodland interior, woodland edge, urban green space, and urban grey space. The probability of recording *P*. *pygmaeus* (relative to recording *P*. *pipistrellus*) within each point count location was included as the response variable. Habitat (e.g. woodland interior) was included in the model as a fixed factor, whereas ‘site’ was used as a random factor (to account for pseudoreplication within sites). Date and temperature were included as covariates.

## Results

### Woodland stand survey

We recorded a total of 2,364 bat passes during a total of 124 hours of surveys. Bats were recorded within all but one of the 31 woodlands surveyed. We recorded a total of 1,584 *P*. *pygmaeus* passes (67% of all bat passes) in 28 of the woodlands, and 642 (27%) *P*. *pipistrellus* passes in 23 woodlands. A further 68 pipistrelle passes were recorded however these could not be classified to species level. Additionally, we recorded 69 *Myotis* spp. bat passes within seven woodlands and one *P*. *nathusii* pass. Both of these taxa were found in an insufficient number of sites for robust statistical analysis and were therefore excluded from further analysis.

**Fig 2:**
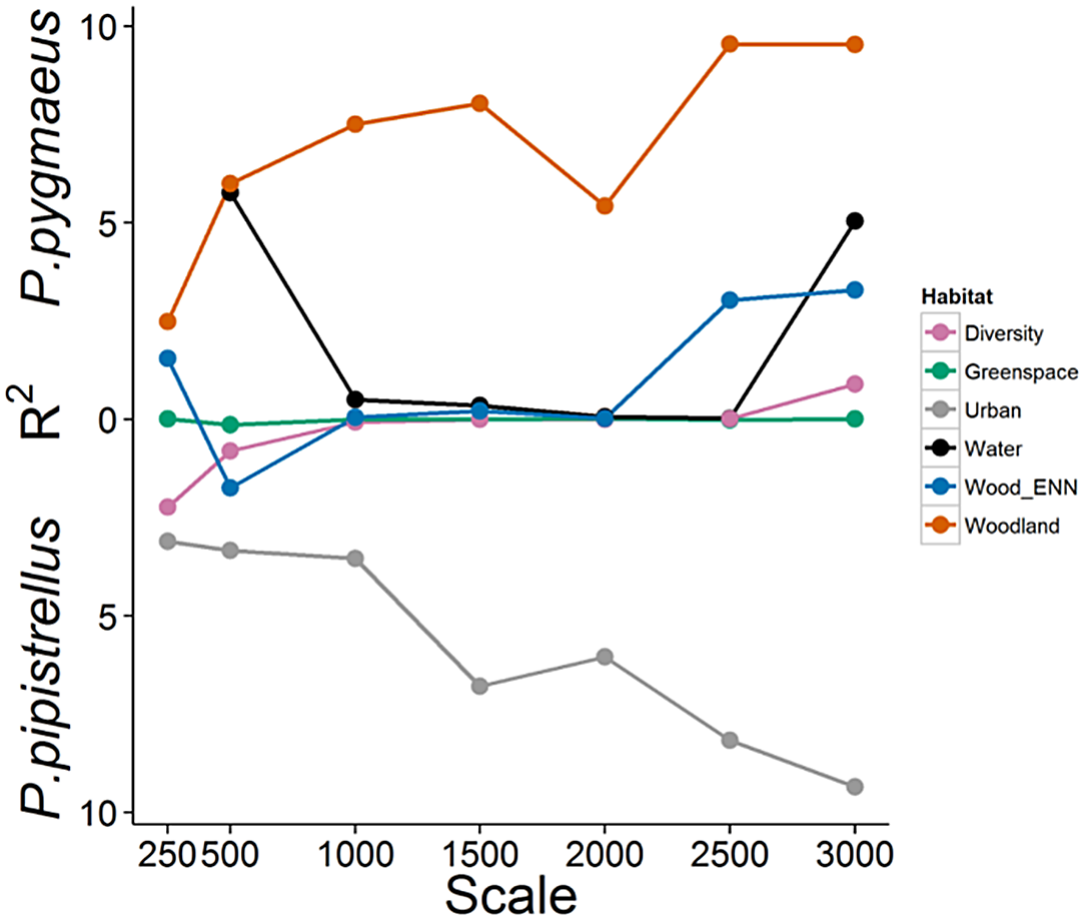
Differential responses to the urban landscape at a variety of spatial scales by cryptic bat species. R^2^ values obtained from GLMM models with binomial error distribution comparing the percentage of landscape covered by each biotype type at a variety of spatial scales to the probability of recording *P*. *pygmaeus* relative to *P*. *pipistrellus* in fragmented urban woodland. The position of the R^2^ values along the y-axis reflect the direction of the parameter estimates; hence R^2^ values in the upper half of the graph refer to a landscape metric that is associated with an increased probability of detecting *P*. *pygmaeus*, whilst R^2^ values in the lower half of the graph refer to a landscape metric that is associated with an increased probability of detecting *P*. *pipistrellus*.

In the results described below it should be noted that significant variables derived from the bat GLMMs indicate a differential response between the species to site or landscape characteristics; variables which are similarly influential for both species will not therefore be statistically significant in these models. Preliminary landscape analysis identified the proportion of grey space in the surrounding 3 km as the key landscape predictor (i.e. highest R^2^ value; Fig 2) which was then incorporated into the final model.

**Fig 3:**
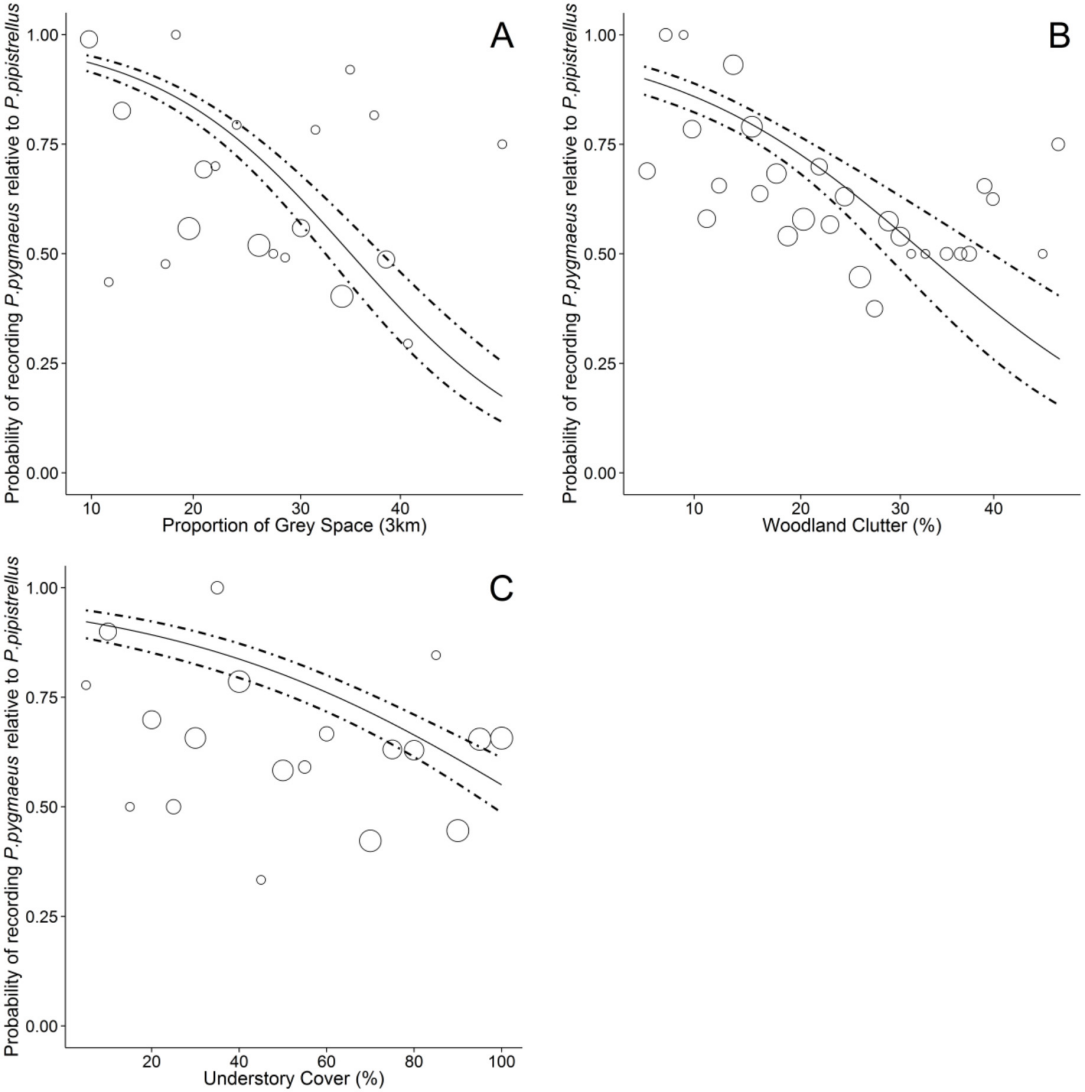
Differential responses to urban woodland and the surrounding landscape by cryptic bat species. Estimated probability of recording *P*. *pygmaeus* relative to *P*. *pipistrellus* in fragmented urban woodland. Dashed lines indicate 95% confidence intervals. Original data on the proportion of *P*. *pygmaeus* passes are superimposed as grey circles with diameter proportion to the total number of *P*. *pygmaeus* passes recorded.

The importance of woodland vegetation characteristics and the surrounding landscape differed between *P*. *pygmaeus* and *P*. *pipistrellus* (Table 2). The proportion of grey space in the surrounding 3 km had the largest effect size and a negative influence on the probability of recording *P*. *pygmaeus* relative to *P*. *pipistrellus*. Based on the estimated coefficients in Table 2, the predicted probability of recording *P*. *pygmaeus* was 0.93 (0.91–0.95) in woodlands surrounded by only a low proportion (10%) of grey space, whilst there was an equal probability of recording either *P*. *pygmaeus* or *P*. *pipistrellus* in woodlands surrounded by moderate levels of grey space (30%; Fig 3A). In woodlands surrounded by high levels of grey space (45%), the predicted probability of recording *P*. *pygmaeus* relative to *P*. *pipistrellus* was 0.17 (0.12–0.25). Woodland clutter had the largest effect size of the vegetation characteristics we assessed. There was a high probability of recording *P*. *pygmaeus* in woodlands with low (10%) clutter (0.86; 0.82–0.89), whilst in densely cluttered woodlands (40%) the probability of recording *P*. *pygmaeus* relative to recording *P*. *pipistrellus* fell to 0.37 (0.26–0.50; Fig 3B). Similarly, the probability of recording *P*. *pygmaeus* in woodlands with low understory cover (20%) was 0.89 (0.85–0.92), whilst in woodlands with continuous understory cover (100%) there was a similar probability of recording either *P*. *pygmaeus* (0.55; 0.49–0.61) or *P*. *pipistrellus* (0.45; 0.39–0.51; Fig 3C). Additionally the probability of *P*. *pygmaeus* decreased in woodlands with a high average tree basal area, however the effect size was relatively small (Table 2).

**Table 2:** Parameter estimates and likelihood ratio tests of the GLMM for the relative proportion of *P*. *pygmaeus* passes to *P*.*pipistrellus* passes in urban woodland.

Fixed effects	Estimate (± SE)	Log Likelihood	χ2	df	*p*
**Intercept**	1.53 ± 0.57				
**Date**	-0.35 ± 0.44	-165.99	0.58	1	0.45
**Temperature**	0.28 ± 0.46	-165.87	0.35	1	0.56
**Tree basal area**	-0.26 ± 0.09	-170.86	10.3	1	0.001**
**Tree species richness**	0.02 ± 0.14	-165.71	0.02	1	0.90
**Proportion grey space (3km)**	-1.05 ± 0.41	-168.71	6.01		0.01*
**Woodland canopy cover**	-0.13 ± 0.14	-166.15	0.90	1	0.34
**Woodland clutter**	-0.73 ± 0.16	-176.11	20.8	1	<0.001***
**Woodland shape**	-0.63 ± 0.45	-166.62	1.84	1	0.17
**Woodland size**	0.07 ± 0.43	-165.71	0.03	1	0.85
**Woodland type**	-0.92 ± 0.86	-166.29	1.18	1	0.28
**Woodland understory**	-0.69 ± 0.14	-178.74	26.1		<0.001***
Shape * Size	0.11 ± 0.75	-166.82	2.28	2	0.52

The model was run to calculate the probability of recording a *P*. *pygmaeus* pass relative to *P*.*pipistrellus*; hence positive estimates indicate an increased probability of detecting *P*. *pygmaeus* and negative estimates indicate an increased probability of detecting *P*. *pipistrellus* with a given explanatory variable. Test statistics were derived from the deletion of each term from the full model (for the 2-way interaction) and from the model with main effects only (main effect terms).

Significance codes:

‘***’ p<0.001,

‘**’ p<0.01,

‘*’p≤0.05.

### Urban matrix survey

**Fig 4:**
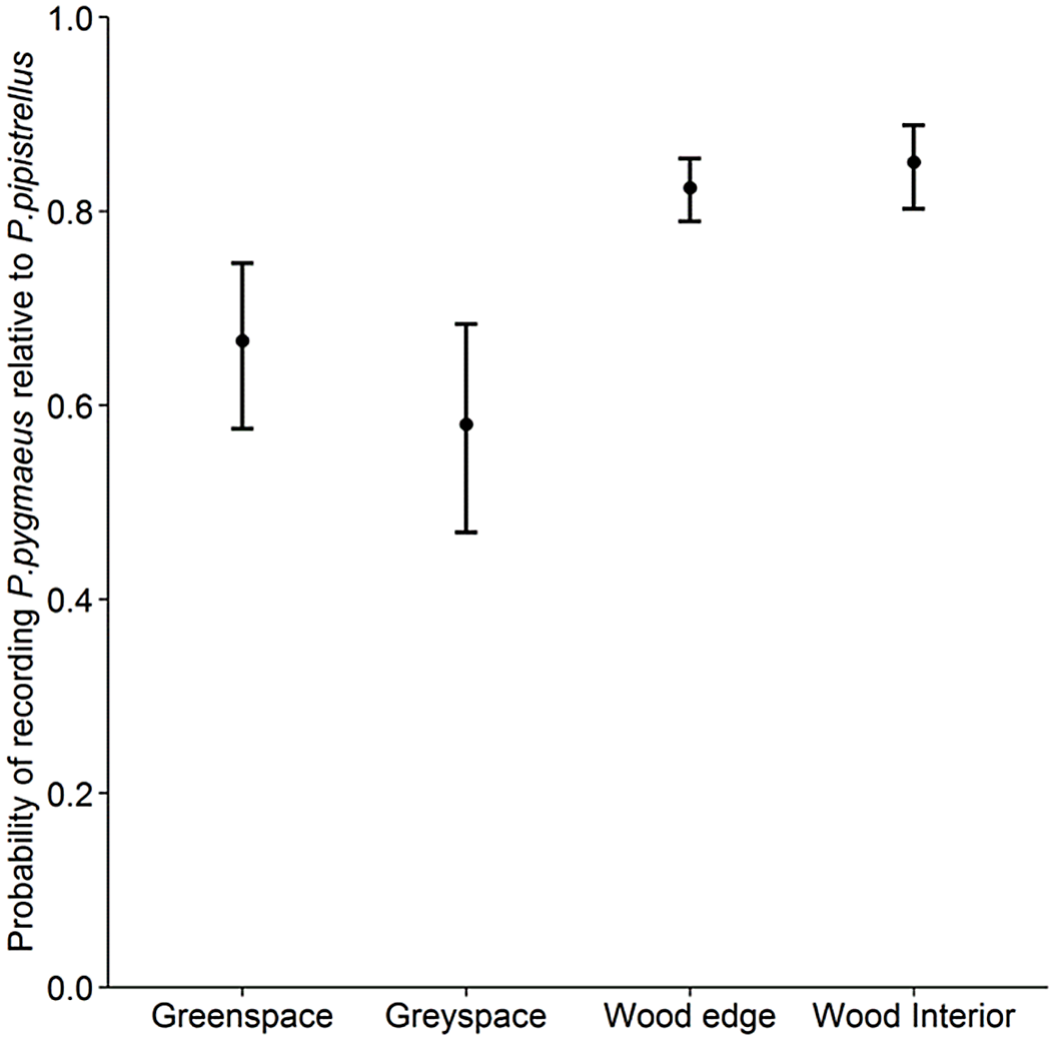
Differential habitat use in the urban matrix by cryptic bat species. Boxplot showing the estimated probability of recording *P*. *pygmaeus* relative to recording *P*.*pipistrellus* in the urban matrix. The upper and lower whiskers show 95% confidence limits. Fitted values by GLMMs are used.

We recorded a total of 260 *P*. *pipistrellus* passes and 701 *P*. *pygmaeus* passes within the four habitat types. The probability of recording *P*. *pygmaeus* relative to recording *P*. *pipistrellus* was significantly associated with habitat type (χ2 = 20.57, df = 3, p<0.001), and was substantially higher in woodland (interior and edge) than the surrounding urban matrix (Fig 4). There was no substantial difference in the probability of recording *P*. *pygmaeus* (relative to *P*. *pipistrellus*) between urban green space and non-wooded grey space (Fig 4).

## Discussion

Determining the ecological and behavioural mechanisms driving habitat use within the urban matrix is the key to understanding the adaptability of species to urbanisation. In this study we show that even two morphologically similar species can have widely differing responses to fragmented urban woodland and the surrounding urban matrix.

### The response of *P*. *pipistrellus* and *P*. *pygmaeus* to urban woodland vegetation character and patch configuration

Although habitat partitioning between *P*. *pipistrellus* and *P*. *pygmaeus* is known from radio tracking studies (e.g. [41]), these studies have involved relatively small sample sizes and were conducted in non-urban habitats. Whilst previous studies have indicated that habitat partitioning between the two species occurs between habitat types (e.g. [42]), here we show that similar behaviour occurs within habitat types, at a fine spatial scale.

Our results indicate that *P*. *pygmaeus* appear to be using woodlands with low clutter and understory growth relatively more intensely than *P*. *pipistrellus*, despite both species having similar wing shapes and echolocation calls which make them well adapted to foraging along woodland edges and relatively open habitats [43]. These findings support Davidson-Watts & Jones [42] who found that *P*. *pygmaeus* spend less time flying, make fewer foraging bouts but travel greater distances, suggesting that this species has more selective foraging habitats. Conversely, *P*. *pipistrellus* is commonly regarded as a generalist forager [42, 44], and therefore would be expected to be found in a wider range of habitat types. Although it is surprising that *P*. *pipistrellus* are not also using less cluttered habitats, Nicholls and Racey [41] suggested that *P*. *pipistrellus* actively avoid *P*. *pygmaeus* foraging sites (but see [45]). Coexisting species must differ in at least one niche dimension to avoid excessive competition such as using different foraging locations [46]. It is therefore possible that the use of woodlands with high clutter and understory by *P*. *pipistrellus* may reflect the wider, non-selective, use of woodland habitats within the urban matrix to avoid competition. In contrast, *P*. *pygmaeus* may be preferentially selecting those woodlands which offer optimal foraging locations. *Pipistrellus* species are known to also forage above the canopy of closed mature woodland stands [47] which although not recorded in this study, may provide additional or alternative foraging resources for either or both *Pipistrellus* species. Differences in habitat use may also reflect that the diets of the two species differ [48]. Assessing which woodland characteristics determine prey availability may also help explain differential habitat use.

### The response of *P*. *pipistrellus* and *P*. *pygmaeus* to the composition, spatial configuration, and heterogeneity of the surrounding landscape

Regardless of the spatial scale surrounding the woodland (250 m to 3 km) we found that the probability of recording *P*. *pygmaeus* relative to *P*. *pipistrellus* was greater when the landscape contained a high proportion of woodland and a low proportion of urban grey space. This supports previous studies identifying *P*. *pipistrellus* as a generalist species [32, 49] which can tolerate moderate levels of urbanisation [19]. Similarly, the proportion of grey space in the surrounding 3 km was the strongest predictor of which of the two species would be recorded. The underlying cause as to why *P*. *pipistrellus* is better able to adapt to the urban landscape is unknown although the lower frequency of its echolocation call may aid adaptability to cities as species with lower calls are better able to use open habitats and access a wider spectrum of habitats [50]. Alternatively, *P*. *pipistrellus* may have the greater behavioural capacity to adapt to exploit the urban landscape, for example using buildings and planted tree lines as paths for courtship flights and territory boundaries [51]. It is surprising given the strong association between *P*. *pygmaeus* habitat preferences and waterbodies [44] that the composition of water in the landscape was not a significant predictor of differences in habitat use between the two species. However, urban waterways are frequently used by both species (Lintott et al. unpublished data) and it is likely that the continuous nature of urban waterways is facilitating the movement of both species through the urban matrix. Additionally, in contrast to alternative biotope types (e.g. green space or grey space) there was relatively little variability between sites in the composition of freshwater in the surrounding landscape (S1 Table), which may have masked any differing habitat use as consequence of freshwater.

### Differences between *P*. *pipistrellus* and *P*. *pygmaeus* in habitat selection within the urban matrix

The higher adaptability to the built landscape by *P*. *pipistrellus* relative to *P*. *pygmaeus* is further supported by the extent to which this species was recorded in a variety of habitat types contained within the urban matrix. In contrast, *P*. *pygmaeus*, relative to *P*. *pipistrellus*, predominantly used woodland edge and interior habitats; foraging locations that both species are well adapted for. This strengthens Nicholls and Racey’s [41] findings that *P*. *pipistrellus* appear to actively avoid *P*. *pygmaeus* foraging sites resulting in differential habitat use. Within the urban matrix, this may transpire as *P*. *pipistrellus* appearing to using a wider range of habitats, thereby giving the impression that it is a generalist. Similarly *P*. *pipistrellus* may be commuting further to use those woodlands which offer suitable foraging resources but are surrounded by sufficient grey space to deter *P*. *pygmaeus*.

### Conservation implications

Understanding how species respond to urbanisation is critical in identifying priority species which may require conservation effort. Categorising species as either ‘urban avoiders’, ‘urban utilizers’ or ‘urban dwellers’ appears a convenient way of achieving this [5]. Bats are often categorised in this manner based upon their morphological traits (e.g. [50, 52]), however our results show that habitat use differs between species which are morphologically very similar [53, 54] suggesting that such differences may be a weak indication of ecological differences between taxa [41, 55]. Similarly, using species presence as an indication of adaptability to the built landscape should be treated cautiously prior to assessing if adaptability to urbanisation is sex dependent [56], or if species in urban landscapes largely consist of sink populations. If *P*. *pipistrellus* are using the urban ecosystem as a means of avoiding competition with *P*. *pygmaeus* it may be that they are not so much exploiting the urban landscape but using it out of necessity.

## Conclusions

The complexity of understanding species-specific responses to urbanisation makes identifying priority species for conservation action difficult. Here, we show that attempts to use morphological traits as a means of categorising species into the likelihood of them adapting to urban locations are problematic, as even two sympatric and cryptic species can respond differently. *P*. *pygmaeus* appear to be using less cluttered woodlands whilst *P*. *pipistrellus* appear to be adapting a generalist foraging behaviour using, often cluttered, woodlands surrounded by relatively high levels of urban grey space; this may be a result of differential habitat use to avoid competition.

## Supporting Information

S1 Table The composition of the landscape surrounding the 31 urban woodlands surveyed throughout central Scotland in 2011. (DOCX)
