# Supplementary material for: Differential Responses to Woodland Character and Landscape Context by Cryptic Bats in Urban Environments
Source: PLoS One. 2015 May 15;10(5):e0126850. doi: 10.1371/journal.pone.0126850 (PMC4433195; doi:10.1371/journal.pone.0126850)
Supplement: S1 Table — (DOCX) [file pone.0126850.s001.docx]

| Scale (m) | % of habitat type within landscape (Mean ± SD) | | | |
| --- | --- | --- | --- | --- |
|  | Woodland | Greenspace | Grey space | Freshwater |
| 250 | 26 ± 16 | 47 ± 14 | 24 ± 12 | 1 ± 1 |
| 500 | 14 ± 9 | 51 ± 11 | 29 ± 9 | 1 ± 1 |
| 1000 | 10 ± 6 | 50 ± 8 | 30 ± 8 | 1 ± 1 |
| 1500 | 10 ± 5 | 48 ± 7 | 30 ± 8 | 1 ± 1 |
| 2000 | 10 ± 5 | 45 ± 8 | 29 ± 8 | 1 ± 1 |
| 2500 | 10 ± 4 | 45 ± 8 | 27 ± 8 | 1 ± 1 |
| 3000 | 10 ± 4 | 45 ± 9 | 26 ± 9 | 1 ± 1 |
